# The mediating role of pro-environmental attitude and intention on the translation from climate change health risk perception to pro-environmental behavior

**DOI:** 10.1038/s41598-024-60418-7

**Published:** 2024-04-29

**Authors:** Tao Shen, Irniza Binti Rasdi, Nor Eliani Binti Ezani, Ong Tze San

**Affiliations:** 1 Department of Environmental and Occupational Health, Faculty of Medicine and Health Sciences, University of Putra Malaysia, 43400 Serdang, Selangor Malaysia; 2 Clinical Laboratory, Jincheng People’s Hospital, Jincheng, China; 3 School of Business and Economics, University of Putra Malaysia, Serdang, Selangor Malaysia

**Keywords:** Pro-environmental behavior, Climate change health risk perception, Pro-environmental attitude, Pro-environmental intention, Hospital worker, Behavioural ecology, Environmental social sciences

## Abstract

Climate change is a serious environmental issue appearing in China. As a public service institution operating around the clock, the negative impact of hospitals on the environment is evident, promoting their workers’ pro-environmental behavior (PEB) through increasing climate change health risk perception (CHRP) is an effective method to protect the environment and achieve sustainable development. This study investigates how CHRP shapes pro-environmental attitude (PEA), pro-environmental intention (PEI), and pro-environmental behavior (PEB) among hospital workers. Using structural equation modeling (SEM) to determine the chain of causation from CHRP to PEB among hospital workers. The result shows that CHRP positively affects PEA and PEI, and PEI positively affects their PEB. In addition, although CHRP has no significant direct effect on PEB, it can play a crucial indirect effect through the mediating role of PEI. Moreover, the result of multiple regression shows that there are significant differences regarding PEA, PEI, and PEB.

## Introduction

Climate change is an extremely serious issue threating the sustainable development of human society^1^. It is acknowledged that human behaviors, such as driving cars and using energy, play the crucial role leading to climate change, and meanwhile can result in its mitigation^2^. In China, environmental issues including pollution, extreme weather, and energy crises are urgent due to the rapid development of the economy, numerous researchers have probed to settle these issues^3–6^. Focusing on individual PEB and its influencing factors is prospective to improving the relatively hostile environment^7,8^. Hospital workers’ health practices take core responsibilities in ensuring human health by providing diagnosis, treatment, and management diseases^9^. Additionally, it is noticeable that the hospital makes an excellent contribution to carbon emissions by energy consumption and delivering care^10–12^. As a consequence, we examined Chinese hospital workers’ PEB and influencing factors to promote their actual PEB in medical practice.

In the process of modernization, although China has carried out large-scale pollution prevention and ecological environment protection strategies, the average temperature has increased by 0.62 ℃, the average precipitation has decreased by 5%, and natural disasters such as droughts and floods still occur frequently according to the Bulletin on the State of China’s Ecological Environment. (https://www.gov.cn/govweb/lianbo/bumen/202305/content_6883708.htm). To deal with severe environmental issues, the Chinese government has commenced a series of column projects, for example, advancing the defense of the blue sky, distributing national climate change adaptation strategies, and prohibiting burning straws^13^. The role of environmental protection played by these projects is obvious and positive. Nevertheless, strategies and measures responding to environmental issues are very limited, and the effort at the government level only is far from enough^14^. Dudney, Willing^15^ also suggested that climate change can lead to specific susceptibilities to various diseases, this is even worse for hospital workers who are already overloaded. However, the fact is that hospital workers habitually neglect the importance of their PEB and are not willing to take related actions such as reducing the use of disposable gloves and selecting local medical devices, which exacerbates the deterioration of the environment and causes more severe health problems^16^. Hence, finding an effective approach to promote hospital workers’ PEB has been commenced by more and more scholars.

For hospital workers, their PEB during medical practices is various, for instance, the disposal of pharmaceutical waste^17^, the management of electronic waste^18^, and the pretreatment of medical wastes^19^. Additionally, environmental protection is directly related to human physical and mental health by reducing pollution, protecting natural resources, maintaining ecological balance, and adopting climate change prevention and control measures^20^. The improvement of overall health can in turn reduce the workload of hospital workers, and increase their job satisfaction and happiness^21^. In this respect, the study of PEB at hospital is important because it does not only concern hospital workers’ PEB but also makes huge contributions to their satisfaction and human health. This raises the question-what are influencing factors of their PEB and how to enhance actual PEB? PEB is defined as a kind of behavior selected carefully by individuals that minimizes the adverse effect of human behavior on the environment and improves environmental quality as much as possible^22^. Numerous studies applied different theories suggest that PEB is affected by many factors, such as gender^23^, environmental attitude and intention^24^, and environmental knowledge^25^. These studies try to clarify some influencing factors of PEB in the household or other workplaces, however, hospital workers’ health risk perception of climate change and the potential mechanisms between their CHRP and PEB have not been elucidated thoroughly. To plug the research gap, we develop the conceptual framework and explore the relationship between hospital workers’ CHRP and their PEB based on the theory of planned behavior (TPB)^24^, the theory of attitude-behavior-context (ABC)^26^, and the theory of information-attitude-behavior (IAB)^27^.

In addition, considering the particularity of hospital workers, the mediating role of their PEA and PEI are examined to further explain the relationship between CHRP and PEB. The rationality of selecting hospital workers’ PEA and PEI as mediating factors is that, compared with other occupations, workers at hospital are more autonomous in their decision-making progress due to their expert knowledge and power, which makes PEB especially arduous at hospital^28^. In addition, PEB is recognized as an influential factor in terms of workers’ well-being that is a positive emotional state characterized by satisfaction, joy, and overall positivity^29^. Attitude and intention, likewise well-being, are subjective judgments and individualized experiences^30^. Although the reality is that the hospital exerts obvious adverse effects on the environment through energy consumption, transportation, and product disposal in the process of preventing, treating, and healing diseases, their workers usually ignore these and regard environmental protection as others responsibility^31^. To change their subjective attitude and intention about environmental issues is necessary for adopting PEB. Therefore, it is important to examine how the PEA and PEI of hospital workers affect the relationship between their CHRP and their PEB.

The rest of this paper is organized as follows. In “Theoretical background and hypotheses” section reviews related literature and proposes research hypotheses. In “Research design” section describes specific research methods. In “Data analysis and hypothesis testing” section is research results. Discussion and conclusions are presented in “Discussion” section and “Conclusion” section.

## Theoretical background and hypotheses

### Pro-environmental behavior

Pro-environmental behavior (PEB) refers to a series of behaviors that are related to obtaining materials and energy from the environment and altering the structure of ecosystems in an environmentally friendly way^32^. The domain of PEB comprises recycling including reusing paper, plastic, and containers, saving resources including energy and water, using public transportation, and properly disposing non-recyclable waste^33^. At hospital, PEB is somewhat different such as using less disposable gloves and using less packaging^23^. In addition, using local products rather than imported one is important for environmental protection, because it can relieve the serious pollution resulting from long-distance transport through ships, trucks, and aircrafts^34^. However, aforementioned PEB cannot be widely adopted by hospital workers^35^. The willingness to adopt PEB is mainly affected by the mixture of self-interest motives and pro-social motives^36^. When individuals’ self-interest motives including economic interests, career development, health, and well-being are positively related to their PEB, more PEB may be adopted to maximize their own interests concerning economics and health^37^. For pro-social motives, generally occur when individuals are willing to help others and prevent risks that may threaten human health^38^. Additionally, PEB is also influenced by socio-demographic factors such as gender, length of employment, and employment department.

### Climate change health risk perception and pro-environmental behavior

Risk perception is a subjective judgment about hazardous events and can explain how risk is perceived and how much adverse effect is caused^39^. Risk perception is extremely extensive and complex, it can be quantified and predicted by various factors not only psychological elements including people’s beliefs, attitudes, judgments, and feelings, but also risk communications about how to prevent and deal with them^40^. The environment provides abundant resources to support human survival and development, hence, human health may be threatened tremendously when the natural environment is destroyed^41^. Climate change, as a global environmental issue, has been deemed as the biggest human health threat in the twenty-first century^42^. The disease deriving from environmental risk factors such as air pollution and water pollution is ever-growing, specifically, heat-related illness, the spread of vector-borne diseases, respiratory diseases caused by air pollution, malnutrition, forced migration, and mental health^43^. An unfavorable fact is that the mortality rate attributed to the damaged environment accounts for one-fourth of the total mortality rate^44^.

According to research in environmental psychology, environmental perception takes shape from the process of communication between humans and nature and reflects individuals’ attitude with regard to the environment^45^. Climate change health risk perception (CHRP) points to a way that individuals or organizations perceive the latent and obvious health risks associated with climate change, and then form a subjective judgment of the probability and severity of these events, which consists of the following three primary parts-how to handle information about risks related to climate change, how to perceive these risks, and how to respond to these risks^46^. For hospital workers, CHRP is to identify the health issues caused by climate change and take proactive actions to avoid these events^47^. Hence, CHRP can be a momentous factor to predict whether people are willing to take action to mitigate climate change and address health problems such as anxiety and depression^48^. Nevertheless, risk perception is diverse and unstable since it can appear or disappear varying from different geographical and demographical features^49^. How to increase the health risk perception of climate change is an emerging field that has not yet been fully elucidated. It is not enough to make efforts by governments and public institutions alone, scholars focusing on environmental protection should explore the influencing factor of risk perception and try to clarify the relationship between them. There are several influencing factors of CHRP such as the level of environmental knowledge, personal experiences with extreme weather events, and environmental beliefs and values^50–52^. Additionally, according to social amplification theory, health risk can be magnified in the communication process, ultimately, people’s risk perception is deepened and they are more willing to take effective actions to protect the environment^53^. Overall, understanding how to perceive climate change health risks is important for developing effective methods to promote the adoption of more actual PEB.

Based on the IAB, individuals’ perception has a significant influence on their attitude and certain behavior^54^. When facing diverse health risks, the more menaces are perceived by hospital workers, the more actions are taken to alleviate these risks^54^. In addition, information has a direct influence on individuals’ perception, the information about climate change also can shape individuals’ health risk perception and contribute to the adoption of PEB^55,56^. Every hospital makes huge contributions to environmental issues in the progress of diagnosis, treatment, and management diseases^9^. Moreover, energy consumption and carbon emission of hospital are high due to operating around the clock^57^. At present, numerous regulations and plans focusing on energy saving and emission reduction have been formulated in the healthcare process, for example, using a digital medical record system to reduce the demand for paper, this not only helps save paper, but also reduces energy costs for transferring files^58^. When considering the possible health risk of climate change, hospital workers will regard these risks as the outcome of their medical practice and daily activities, and they will be willing to take more effective measures to minimize these risks and promote everyone’s health. In short, the reason for supporting risk perception research is that it reflects people’s preferences, underlying values, and information concerning risk.

In China, due to its diverse climatic characteristics and unbalanced socio-economic development, climate change poses greater health risks^59^. These risks are perceived by individuals in various ways due to the difference between the working environment and personal experience^60^. However, current studies generally focus on the public or recognized sensitive groups such as farmers^61^, the risk perception of hospital workers is missing, which poses higher risks and threatens human health directly and indirectly^62^. For hospital workers’ CHRP, what differences exist and how to improve it remain confusing. The study of hospital workers’ CHRP is significant, such as decline in climate change related diseases, decrease in medical expenditures, optimization the use of medical resources, and promotion public health^63^. Hence, this study aims to clarify the relationship between CHRP and PEB, and to explore how CHRP affects hospital workers’ PEB. In the light of above discussions, we propose the following hypothesis:

#### Hypothesis 1

Hospital workers’ climate change health risk perception has a significant positive effect on pro-environmental behavior.

The IAB also points out that individuals’ perception has a significant influence on personal attitude and then changes certain behavior^54^. The study of O'Connor, Bard^64^ suggested that individuals with higher risk perception are more positive to deal with environmental issues. Additionally, there is a study that believes the risk perception shaped by information from surrounding people and the internet can change individuals’ attitude positively toward green purchasing behavior^65^.

Dawson^54^ also gave the same conclusion, environment related risk perception can influence indirectly people’s PEB via the mediating role of attitude. In China, the problem of environmental pollution is becoming increasingly prominent with the development of the economy, the public shows a high level of risk perception and a positive attitude to address these issues^66^. Moreover, Ban, Shi^67^ indicated that the more latent risks of climate change such as heat wave are perceived, the more willing appear to mitigate these issues. Based on the same logic, it is reasonable to speculate that hospital workers perceiving higher health risks are more willing to take effective measures to reduce those risks. Thus, the following hypothesis is proposed:

#### Hypothesis 2

Hospital workers’ climate change health risk perception has a significant positive effect on pro-environmental attitude.

According to the TPB, intention is a direct predictive factor for specific behavior, hence, it can serve as a mediator to affect the relationship between other influencing factors and actual behavior^24^. By the same token, the causal relationship between CHRP and PEB may be mediated by intention. Yoon, Jeong^68^ mentioned that risk perception of ocean microplastics plays a crucial role in tourists’ intention toward environmental protection. In addition, a study using the artificial intelligence (AI) voice demonstrates that AI can elicit risk perception and then motivate pro-environmental behavioral intention^69^. As for sustainable consumption behavior, risk perception regarding environmental issues makes significant contributions to the increased behavioral intention through the role of environmental concern^70^. Another study focusing on environmental pollution shows that when local residents perceive more adverse effects of pollution, they show more intention to take measures such as reducing car use to mitigate pollution^71^. The hospital is the main force to deal with adverse health effects from climate change, which makes the study focusing on hospital workers’ PEB important^72^. For hospital workers, several studies have clarified the relationship between their risk perception and intention to protect the environment, which presents a positive correlation^67,73,74^. Hence, in this study, we can predict that the more health risk perceived by hospital workers, the more positive intention can form toward environmental protection. The following hypothesis is proposed:

#### Hypothesis 3

Hospital workers’ climate change health risk perception has a significant positive effect on pro-environmental intention.

### Pro-environmental attitude

Attitude is a subjective and psychological inclination of an individual toward a specific person, event, or concept, which can affect individuals’ perception, emotion, and behavior^75^. Pro-environmental attitude (PEA) refers to a strong willingness to adopt PEB to protect the environment and achieve sustainable development, it is relatively stable and comprises three main aspects: cognitive composition, emotional composition, and behavioral tendency composition^76^. Therefore, attitude, as a critical variable to predict behavior, is incorporated into multiple theories to understand human behavior, such as the TPB (Ajzen, 1991), the IAB^27^, and the ABC^26^. Specifically, TPB has become an ideal and powerful instrument to explain and predict behavior in the field of physical activity, consumer behavior, and privacy protection^77–81^. At present, many instruments have been applied to predict and measure individuals’ attitude, among them, the most widely used is the new ecological paradigm (NEP) proposed by Riley E. Dunlap in 1980^82^. Based on ecological principles, the aim of NEP is to understand individuals’ perception and attitude of environmental issues. The complete questionnaire of NEP contains several facets such as balancing the ecosystem, negating anthropocentrism, restricting the use of natural resources, and reducing the possibility of an eco-crisis^83^.

In terms of environmental protection, a great number of studies have a special focus on individuals’ attitude towards the environment and draw a conclusion that these attitudes contribute to the adoption of PEB^84–86^. Generally, the environmental attitude is divided into two facets: attitude towards the overall environment or specific parts such as water and soil, attitude towards pro-environment behavior such as recycling and energy conservation, which can be influenced by multitudinous factors from the level of environment, social, and individual^87^. However, there is a noteworthy “attitude-behavior” gap, an example is the study focusing on green purchasing, consumers realize the severity of environmental issues and are concerned about these, however, they do not display actual green purchasing behavior^88^. Redondo and Puelles^89^ also pointed out a significant attitude-behavior gap in the field of unhealthy diets including fast food, alcoholic beverages, and pre-cooked meals, the lack of self-control is a reasonable cause to explain this gap. In addition, this gap also appears in tourism, tourists’ are willing to take effective behaviors to protect the environment, however, it has not become an actual PEB^90^. Even so, using PEA to explain and predict actual PEB is feasible. Most of current studies regard the TPB as a theoretical basis to explain the relationship between attitude and behavior, it is worth noting that this interaction is indirect, specifically, the attitude plays a direct role in intention, and then contributes to particular behavior^24^. At hospital, the TPB is also applicable, and Widianto, Kautsar^19^ found that hospital workers’ PEA can forecast intention related to PEB. However, the ABC and IAB demonstrate a direct link between attitude and behavior, as for hospital workers, whether this direct relationship exists needs more studies to elucidate. In the light of the above discussions, we propose the following hypothesis:

#### Hypothesis 4

Hospital workers’ pro-environmental attitude has a significant positive effect on pro-environmental behavior.

### Pro-environmental intention

Intention, as a direct precursor to behavior, is a predictive psychological state that reflects individuals’ plans and actual behavior. The formation of intention is sophisticated, and individual beliefs, attitudes, social pressure, and self-efficacy can influence this process^91^. Pro-environmental intention (PEI) is individuals’ willingness to adopt behavior related to environmental protection, it is a collection of intrinsic motivations for taking PEB^92^. More and more scholars have dedicated to applying the TPB to gain a deep insight of PEB such as the reduction of haze pollution^93^, the utilization of public transport^73^, and the classification of waste^94^. The TPB indicates that the intention is a direct predictor to predict the performance of a specific behavior, and the stronger the intention, the more likely individuals are to execute the behavior^24^. However, the formation of intention is not simple, Ajzen^77^ demonstrated that individuals’ attitude, subjective norm, and perceived behavioral control towards environmental issues can affect their intention and eventually promote or impede PEB.

According to the TPB, attitude towards behavior is a remarkable predictor that can explain and promote behavioral intention (Ajzen, 1991). The study of Kalafatis, Pollard^95^ also clearly determines the appropriateness of using TPB to explain intention. Nowadays, relevant research suggests that PEI is strongly or moderately related to PEB. For example, Widianto, Kautsar^19^ demonstrated that individuals possessing the intention to sort and recycle wastes adopt more actual PEB such as waste disposal. Shimoda, Hayashi^96^ suggested that PEI can further promote or hinder individuals’ green purchasing behavior. At hospital, human health is an important driving factor of PEI and PEB since ensuring human health is the core responsibility of medical workers^97^. However, the existence of intention-behavior gap is nonnegligible, what can explain this gap is numerous barriers including lack of environmental knowledge, lack of role models, and lack of economic support impede the adoption of PEB^98^. How to overcome aforementioned difficulties and make contributions to the implementation of PEB become the focus of future research. Even so, the TPB is still an ideal theory to explain and speculate the relationship between hospital workers’ PEI and PEB. In the light of above discussions, the following hypothesis is proposed:

#### Hypothesis 5

Hospital workers’ pro-environmental intention has a significant positive effect on pro-environmental behavior.

**Figure 1:**
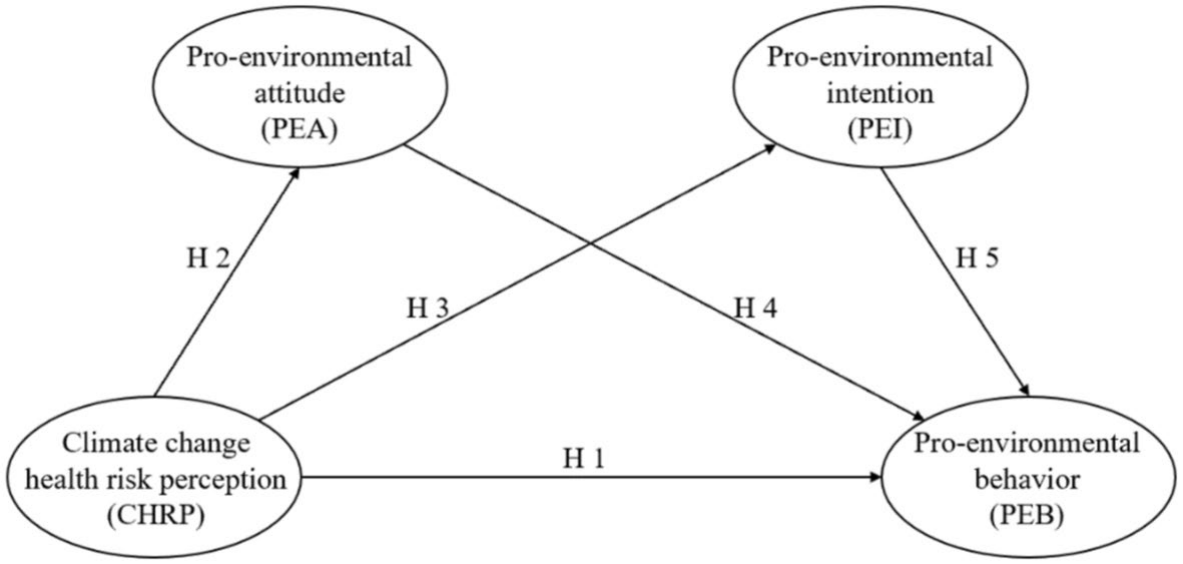
Conceptual framework.

Figure 1 depicts our conceptual framework with hypotheses.

## Research design

### The research population and sample

For the sake of obtaining an overall understanding of hospital workers’ health risk perception resulting from climate change, we conducted a questionnaire survey through simple random sampling. All experimental protocols were approved by the Ethics Committee for Research involving Human Subjects of University Putra Malaysia (JKEUPM), and the reference number is JKEUPM-2023–567. All experimental methods were performed in accordance with the relevant guidelines and regulations, and the informed consent was obtained from each participant. The target population of this study was workers at hospital in Shanxi Province, China, and the research period was from October to November 2023. The regional characteristics and current economic development status of Shanxi Province were the reasons for selection. Specifically, Shanxi Province, located in central China, has a temperate, continental, monsoonal climate with four distinct seasons, which contributes to the susceptibility of this region^99^. Additionally, burning coal is the main source of energy in Shanxi Province, hence, air pollution and greenhouse gas release are increasingly severe^100,101^. Meanwhile, Shanxi’s relatively backward economic development cannot deal with these environmental issues and can cause more serious health problems^100,102^. Therefore, we chose Shanxi Province to conduct this research. In addition, considering the differences in regional differences in climate, Datong, Taiyuan, and Jincheng were selected as research sites.

To ensure the accuracy and representativeness of the samples, we calculated the sample size using OpenEpi (https://www.openepi.com/Menu/OE_Menu.htm). According to the statistics of Shanxi provincial government, the population size of hospital workers is 281,533 at the end of 2021, which requires 384 participants at a minimum (95% confidence level). In formal study, 10 hospitals were randomly selected from each city, and 20 questionnaires were distributed to each hospital. During the research period, 600 questionnaires were distributed, and 543 valid questionnaires were obtained.

### Variable measurement

#### Climate change health risk perception

Based on the study of Wang, Jiang^103^, Hathaway and Maibach^104^, Thaker, Richardson^105^, and others, CHRP was assessed using four items (1. I will be at risk to get physical harm from extreme weather events such as severe storms or flooding; 2. I will be at risk to get health stroke from extreme heat waves; 3. I will be at risk to get asthma and/or other lung diseases due to increasingly severe pollution; 4. I will be at risk to get diseases carried by insects). This four-item measure has two main aspects: physical harm and mental health. According to hospital workers’ responses to these four items, we measured the level of their CHRP using a seven-point Likert scale ranging from 1, “strongly disagree”, to 7, “strongly agree”.

#### Pro-environmental attitude

PEA was assessed using four items (1. I feel responsible to save resources whenever possible; 2. I feel responsible to reduce energy consumption whenever possible; 3. I feel responsible to consider the environmental effects of my work whenever possible; 4. I feel responsible to find new ways to improve the environmental performance of the hospital) extracted from the New Ecological Paradigm (NEP) scale^106^ and the Environmental Attitudes Inventory (EAI)^107^. Hospital workers were required to self-report these items using a seven-point Likert scale ranging from 1, “strongly disagree”, to 7, “strongly agree”.

#### Pro-environmental intention

To measure individuals’ PEI, related items should include clear commitments to environmental actions. In the current study, the measuring of hospital workers’ PEI was developed based on the study applied the theory of planned behavior^108,109^. The measurement of PEI comprised four items (1. I intend to reduce the use of disposable plastic products such as disposable gloves and bottles; 2. I intend to reduce energy consumption such as turning off lights and saving water; 3. I intend to educate patients how to take environmental protection measures; 4. I intend to join environmental organizations and encourage colleagues to adopt pro-environmental behaviors), and hospital workers reported these items using a seven-point Likert scale ranging from 1, “strongly disagree”, to 7, “strongly agree”.

#### Pro-environmental behavior

Recycling and energy saving are two main behavioral categories in the field of environmental protection^18^. According to the study of Lamm, Tosti-Kharas^110^ and Deng, Cherian^18^, four items were applied to measure the actual PEB (1.I recycle my plastic bottles, cans, and other containers; 2. I use scrap paper for notes instead of fresh paper; 3. I turn off the lights in a vacant room; 4. I power down all desk electronics at the end of the day). Hospital workers reported their behaviors via a seven-point Likert scale ranging from 1, “strongly disagree”, to 7, “strongly agree”, and the total score indicated the level of actual PEB.

#### Control variables

According to previous research, gender^111^ and length of employment^112^ have certain impacts on actual PEB. Due to the particularity of hospital department classification, the employment department should also be taken into consideration. Hence, we regarded these three variables as control variables in this study. The specific content is shown in Table 1.

**Table 1:** Demographic and work profile.

Respondents’ characteristics	Items	Frequency (N = 543)	Percentage (%)
Gender	Male	225	41.4
	Female	318	58.6
Length of employment	5 years or below	112	20.6
	6–15 years	123	22.7
	16–25 years	105	19.3
	26–35 years	102	18.8
	36 or above years	101	18.6
Employment department	Emergency department	85	15.7
	Surgery department	138	25.4
	General medicine department	88	16.2
	Traditional Chinese medicine department	98	18.0
	Medical technology department	114	21.0
	Others	20	3.7

### Analytical strategies

#### Reliability test

To ensure the stability and reproducibility of the questionnaire design, we conducted the reliability test. We used SPSS 26.0 to test the reliability of each item in questionnaire by Cronbach’s Alpha coefficient ranging from 0 to 1. The result of Cronbach’s Alpha coefficients was 0.859, 0.864, 0.831, and 0.830 respectively (Table 2). All results were greater than 0.7, which indicated that the questionnaire had good reliability.

**Table 2:** Convergent validity and reliability.

Constructs	Items	Standardized factor load	AVE	CR	Cronbach’s alpha (a)
Pro-environmental attitude (PEA)	PEA 1	0.887	0.7791	0.9461	0.859
	PEA 2	0.811			
	PEA 3	0.840			
	PEA 4	0.876			
Pro-environmental intention (PEI)	PEI 1	0.927	0.6565	0.8827	0.864
	PEI 2	0.879			
	PEI 3	0.709			
	PEI 4	0.701			
Climate change health risk perception (CHRP)	CHRP 1	0.775	0.6122	0.8627	0.831
	CHRP 2	0.745			
	CHRP 3	0.731			
	CHRP 4	0.871			
Pro-environmental behavior (PEB)	PEB 1	0.773	0.5867	0.8493	0.830
	PEB 2	0.652			
	PEB 3	0.797			
	PEB 4	0.830			

#### Validity test

Validity is the degree of agreement between measured results and actual results, and can be measured from two aspects: content validity and structural validity. In terms of content validity, most of the items took shape according to prior studies. In addition, the committee of supervisory of the study also reviewed the items, and master’s degree students majoring in translation were invited to certify the Chinese version. As for structural validity, it comprises convergent validity and discriminant validity, which was measured by AMOS 26.0 using confirmatory factor analysis (CFA). As shown in Table 2, factor load values are greater than 0.6, average variance extracted (AVE) values are greater than 0.55, and combined reliability (CR) values are greater than 0.8. All results indicate that the questionnaire has good convergent validity. As for discriminant validity, the diagonal values are the square of average variance, the non-diagonal values are the square of the correlation coefficient, and diagonal values higher than non-diagonal values indicate good discriminant validity. As shown in Table 3, all the values located on the diagonal exceed other values not on the diagonal, indicating the questionnaire has good discriminant validity.

**Table 3:** Discriminant validity.

	AVE	PEA	PEI	CHRP	PEB
PEA	0.78	0.88			
PEI	0.66	0.38	0.81		
CHRP	0.61	0.32	0.41	0.78	
PEB	0.59	0.36	0.64	0.36	0.77

## Data analysis and hypothesis testing

### Model fit test

A satisfied model is the essential prerequisite of using structural equation model (SEM). According to our results from AMOS 26.0, the value of CMIN/DF (2.454) was less than 5, RMSEA (0.024) was less than 0.05, and GFI (0.901), AGFI (0.892), CFI (0.922), IFI (0.923), and TLI (0.919) approached or exceed 0.9. Thus, the model is a “good fitting” model, and can be used to carry out further research.

### Collinearity diagnostic test

Collinearity refers to the situation in which there is a high degree of correlation or linear relationship between independent variables in a regression model, which may cause the distortion of parameter estimation and the failure of the hypothesis test^113^. To identify whether there was collinearity among variables, we conducted linear regression using SPSS 26.0. Variance inflation factor (VIF) and tolerance value were common statistics to denote collinearity. As shown in Table 4, all the value of VIF is less than 10, and tolerance values are less than 1, indicating that there is no multicollinearity among independent variables including PEA, PEB, and CHRP.

**Table 4:** Results of collinearity.

Variable	VIF	Tolerance	Evidence of multicollinearity
PEA	6.024	0.166	No evidence
PEI	6.963	0.144	No evidence
CHRP	1.178	0.582	No evidence

### Structural results

**Figure 2:**
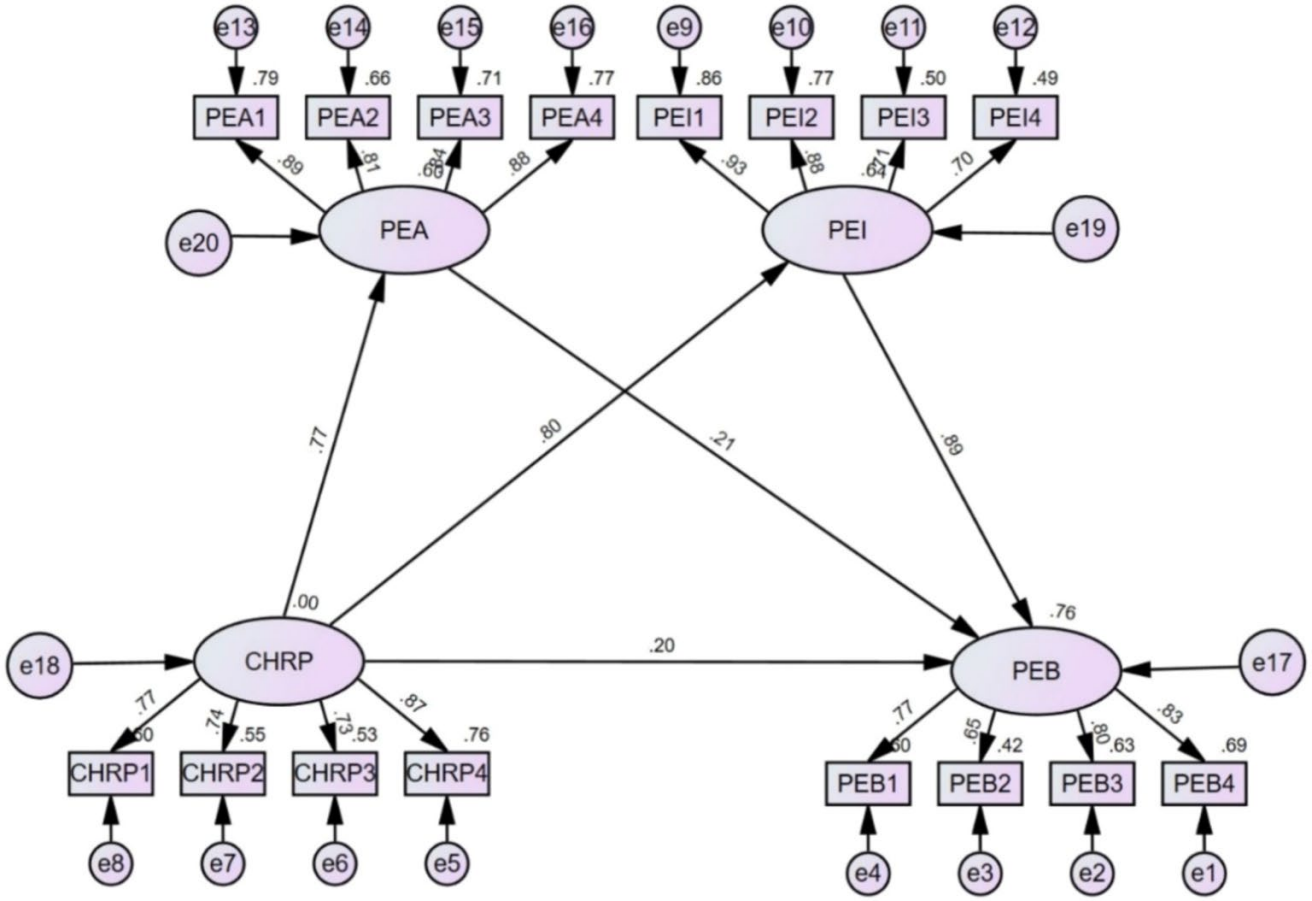
Path diagram of the structural equation model.

Figure 2 is the path diagram of the SEM, and Table 5 is the hypothesis test results. In brief, three hypotheses were supported, and two hypotheses were rejected in this study. Specifically, the standardized coefficient between CHRP and PEA was 0.772 (*P* < 0.001), indicating a positive relationship between them, and hypothesis 2 was supported. The standardized coefficient between CHRP and PEI was 0.803 (*P* < 0.001), indicating a positive relationship between them, and hypothesis 3 was supported. In addition, PEI had a significant positive effect on PEB (standardized coefficient = 0.894, *P* < 0.001), thus, hypothesis 5 was supported. CHRP (standardized coefficient = 0.204, *P* > 0.05) and PEA (standardized coefficient = 0.206, *P* > 0.05) had no significant impacts on PEB, therefore, hypotheses 1 and 4 were rejected. Although there was no significant direct effect between CHRP and PEB, the indirect effect can be achieved by the mediating role of PEI. Hence, we conducted further analysis to test the mediating effect of PEI.

**Table 5:** Results of the structural equation model and hypothesis test.

Path	Standardized coefficient	S.E	C.R	*P*	Hypothesis	Supported
PEB < ---CHRP	0.204	0.256	0.730	0.466	H 1	No
PEA < ---CHRP	0.772	0.140	5.054	***	H 2	Yes
PEI < ---CHRP	0.803	0.155	5.463	***	H 3	Yes
PEB < ---PEA	0.206	0.186	1.110	0.267	H 4	No
PEB < ---PEI	0.894	0.208	3.747	***	H 5	Yes

*P*: *P < 0.05, **P < 0.01, ***P < 0.001.

### Mediating effect analysis

The mediating effect refers that the independent variable influences the dependent variable through the mediating variable, which deepens the understanding of the relationship between variables and reveals the specific pathways from the independent variable to the dependent variable^114^. The causal-step method and the product of coefficients are common approaches to test mediating effects. Recently, more and more controversies come into existence with extensive use of the causal-step method, low statistical efficiency and unable to directly provide confidence intervals are obvious defects of this method^115^. The product of coefficients includes the Sobel test and the Bootstrap method. The Sobel test requires that the sampling distribution of the indirect mediating effect is normal, but in reality, the distribution is asymmetric, which limits the application of the Sobel test when conducting mediating analysis^116^. Therefore, the bootstrap method was used in this study, and the confidence interval did not include 0 indicating the existence of mediating effects.

We conducted the bootstrap analysis using the Process plugin in SPSS 26.0 to analyze the indirect effect of PEI between CHRP and PEB. To achieve this, we chose Model number 4, specified a 95% confidence interval, and selected 5000 as the number of bootstrap samples. As shown in Table 6, the mediating effect of PEI is 0.2235, the direct effect between CHRP and PEB is 0.2615, and the total effect of the independent variable on the dependent variable is 0.4849, in brief, the total, direct, and indirect effect are significant (*P* < 0.001). Additionally, the 95% confidence interval of indirect effect does not contain zero, the conclusion is that PEI can serve as a significant mediator of the relationship between CHRP and PEB. Therefore, although CHRP does not have direct roles in actual PEB, it influences PEB indirectly via the mediating effect of PEI.

**Table 6:** Total, direct, and indirect effects of CHRP on PEB.

Effect source	Effect value	*P*	LLCI	ULCI
Total effect (Total)	0.4849	***	0.4179	0.5502
Direct effect (PEB < ---CHRP)	0.2615	***	0.1967	0.3263
Indirect effect (PEB < ---PEI)	0.2235	***	0.1525	0.2953

*P*: *P < 0.05, **P < 0.01, ***P < 0.001.

### Multiple linear regression analysis

According to previous research, we selected three control variables: gender divided into two groups, length of employment divided into five groups, and department of employment divided into six groups. We used SPSS 26.0 to conduct the inter-group mean comparison, and differences among above variables are shown in Table 7. Specifically, females had higher PEA, PEI, and PEB than males, while there was no significance between their CHRP. As expected, length of employment had noticeable impacts on actual PEB, and people who had worked for 6–15 years adopted more PEB. They also exhibited more PEI and PEA at the same time. However, length of employment did not affect hospital workers’ CHRP. In addition, workers from different departments had different levels of CHRP, PEA, PEI, and PEB, while there was no significant difference in CHRP among the six categories of departments. High PEA, PEI, and PEB appeared in the other group that usually comprised the department of hospital infection management, the department of prevention care, and the department of publicity. It is reasonable to infer that the diversity of primary work responsibilities contributes to these differences.

**Table 7:** Group differences of CHRP, PEA, PEI, and PEB.

Items	CHRP			PEA			PEI			PEB		
	Mean	SD	*P*	Mean	SD	*P*	Mean	SD	*P*	Mean	SD	*P*
Male	20.32	3.50	0.335	20.54	3.56	***	20.01	3.61	***	20.43	3.69	*
Female	20.64	4.09		21.78	3.52		21.25	3.41		21.30	3.35	
5 years or below	20.06	4.28	0.533	21.07	3.73	*	20.24	3.65	***	20.88	3.45	*
6–15 years	20.57	4.28		22.19	3.48		21.55	3.52		21.76	3.48	
16–25 years	20.81	3.70		20.65	3.60		20.54	3.88		20.64	3.41	
26–35 years	20.30	3.27		21.10	3.77		20.17	3.52		20.38	3.55	
36 years or above	20.84	3.40		21.02	3.56		21.00	2.84		20.75	3.62	
Emergency	20.77	3.85	0.195	20.52	3.44	***	20.44	3.59	***	20.91	3.55	*
Surgery	20.27	3.80		21.55	3.68		21.12	3.22		21.26	3.49	
General medicine	21.06	2.75		21.68	2.76		20.70	2.91		21.27	3.09	
Chinese medicine	19.88	3.65		20.42	3.95		19.58	3.91		20.25	4.01	
Medical technology	20.43	3.87		21.04	3.61		20.64	3.56		20.46	3.51	
Others	21.55	3.15		23.85	2.88		23.36	3.84		22.27	2.68	

*P*: *P < 0.05, **P < 0.01, ***P < 0.001.

In this study, the unique predictors of PEB included CHRP, PEI, and PEA. Therefore, together with aforementioned control variables, the regression model consisted of six variables, namely, gender (X1), length of employment (X2), department of employment (X3), CHRP (X4), PEI (X5), and PEA (X6). The prediction equation was given: *Ŷ* = *b1X1* + *b2X2* + *b3X3* + *b4X4* + *b5X5* + *b6X6* + *ε* (Ŷ = PEB, ε = random error). To test this equation, we used the multiple regression analysis method via four models: Model 1-Control variables including gender, length of employment, and department of employment were put into the regression equation; Model 2-Based on Model 1, CHRP as an independent variable was put into the regression equation; Model 3-Based on Model 1 and Model 2, PEI was put into the regression equation; Model 4-Based on aforementioned three models, PEA was put into the regression equation. As shown in Table 8, the standardized coefficient of gender, department of employment, CHRP, PEI, and PEA are all positive: 0.238 (*P* < 0.01), 0.244 (*P* < 0.01), 0.082 (*P* = 0.385), 0.330 (*P* < 0.001), 0.299 (*P* = 0.085), respectively. However, a notable negative value, − 0.222 (*P* < 0.01), exists between length of employment and PEB.

**Table 8:** Results of multiple linear regression.

Variables	Model 1		Model 2		Model 3		Model 4	
	Unstandardized coefficient	Standardized coefficient	Unstandardized coefficient	Standardized coefficient	unstandardized coefficient	Standardized coefficient	Unstandardized coefficient	Standardized coefficient
Intercept	59.651***		34.753***		22.790***		22.004***	
Gender	7.002*	0.245	10.240**	0.358	6.888**	0.241	6.801**	0.238
Length of employment	− 4.272**	− 0.388	− 3.288**	− 0.299	− 2.090*	− 0.190	− 2.449**	− 0.222
Department of employment	3.382**	0.422	2.771**	0.345	1.958**	0.244	1.955**	0.244
CHRP			0.339***	0.469	0.053 (*P* = 0.448)	0.073	0.059 (*P* = 0.385)	0.082
PEI					0.501***	0.621	0.327***	0.330
PEA							0.247 (*P* = 0.085)	0.299
R^2^	0.410		0.608		0.794		0.808	
F	10.650***		17.479***		33.859***		30.080***	
R^2^ change	0.410		0.199		0.185		0.014	
F change	10.650***		22.815***		39.523***		3.101 (*P* = 0.085)	

*P*: *P < 0.05, **P < 0.01, ***P < 0.001.

Table 8 illustrates the multiple regression model of hospital workers’ PEB. The final model (Model 4) including six variables was significant, R^2^ = 0.808 and F (6, 537) = 30.080 (*P* < 0.001), which indicated that about 80.8% of variance in PEB can attribute to variables entered into the regression model. After introducing the variable of CHRP (Model 2), the R^2^ value increased by 0.199, and the change in F was 22.815 (*P* < 0.001), which meant inclusion the CHRP has statistical significance in predicting actual PEB. After increasing the variable of PEI (Model 3), the R^2^ value increased by 0.185, and the change in F was 39.523 (*P* < 0.001), which also proved the necessity of introducing PEI.

## Discussion

From the perspective of Chinese hospital workers, we explore the relationship between CHRP, PEI, PEA and their PEB, as well as whether there is the mediating effect played by PEI and PEA. This study generates several results and makes significant contributions in the field of environmental protection and sustainable development.

First, numerous agendas from the level of national and international have realized the severity of climate change^117^. To reverse the tendency of climate change and improve human health, more and more researches focus on the relationship between CHRP and PEB, additionally, PEA and PEI have been extensively used in previous research. However, most of the research about PEB is conducted in developed countries such as the USA^118^, Sweden^119^, Spain^120^, and Britain^121^, little is known in developing country, which is a deficiency and is obviously magnified in China. Meanwhile, the health effects due to climate change are continuously accelerating in China^122^. The hospital plays a crucial role in protecting health, meanwhile, it also releases a large amount of greenhouse gases leading to climate change through energy consumption, transportation, and product disposal^123^. Hence, this study explored the influencing factor of Chinese hospital workers’ PEB. From the test result of hypothesis 2, we found that CHRP has significant impacts on PEA, which indicates that hospital workers’ understanding of the current environmental situation and their perceptions of the health risk related to climate change are important promoting factors in terms of their environmental attitude. This finding is consistent with the previous research by Bradley, Babutsidze^124^, who compared residents from Australia and France, and claimed that risk perception can indirectly predict environmental behaviors. Additionally, Carducci, Fiore^125^ also indicated that the attitudes towards pro-environmental behaviors are positively related to health risk perception among Italian University Students. Moreover, we found that CHRP also significantly positively affects PEI among hospital workers. This is consistent with the views of Yoon, Jeong^68^ and Ataei, Gholamrezai^126^. Specifically, Yoon, Jeong^68^ indicated that risk perception of ocean microplastics significantly affects pro-environmental behavioral intention of tourists. Ataei, Gholamrezai^126^ demonstrated that farmers’ intention to use green pesticides can be explained by health beliefs including perceived benefits, perceived risk, cues to action, and motivation. According to the test result of hypothesis 5, we inferred that PEI has significant positive impacts on PEB, which conforms to the TPB that suggests behavioral intention is a direct predictor variable that can effectively explain and promote actual behaviors^24^. Chin, Jiang^127^ mentioned that increasing customers’ environmental intention can motivate them to use green cosmetic products. However, Wang and Mangmeechai^128^ suggested that there is an intention–behavior gap in the field of waste sorting and management, Chinese citizens’ environmental intentions have increased, but their actual behaviors may not change accordingly.

Second, based on the test result of hypothesis 1, it indicated that higher CHRP can not necessarily bring about more actual PEB of hospital workers. In previous studies, many scholars have convinced that risk perception has a direct and intimate relationship with environmental behavior. For example, Zeng, Jiang^129^ found that an individual’s pro-environmental behavior can be influenced by environmental risk perception and cultural worldviews. Truelove and Gillis^86^ also found that laypeople’s perceptions in terms of environmental impact and health are tightly related to PEB. However, several studies have shown that the direct relationship between CHRP and PEB is not significant and can be easily influenced by other factors. Yu, Chang^130^ found that social norm is a dominant mediator in regulating the relationship between risk perception and pro-environmental behavior. Maartensson and Loi^131^ pointed out that risk perception is positively associated with behavioral willingness that is positively associated with pro-environmental behavior, this indirect effect between risk perception and pro-environmental behavior is significant. Hence, it is reasonable to claim that individuals’ CHRP alone is unable to fully transform into actual PEB, the top priority is to find more variables that can strengthen or weaken this relationship. Different individuals have different perceptions when encountering the same risks^132^. Hospital workers are regarded as guardians of human health, more and more scholars have put their attention to clarify the obstacles and stimuli behind their health risk perception and environmental behavior, which includes demographic characteristics, cognition, emotion, and culture^19,133–135^. Additionally, hospital workers are not well informed about the health risks associated with climate change^62^. Therefore, CHRP cannot contribute to pro-environmental behavior for every worker. At hospital, PEB is especially arduous since hospital workers have great autonomy when they make a decision about whether to adopt a specific behavior according to their knowledge, experience, and power^28^. Although excellent greatness has been achieved in recent years, individuals from all walks of life still need to improve their CHRP. Like other occupations, hospital workers spend a large chunk of time at workplaces, and their CHRP cannot always lead to actual PEB. Notwithstanding, the indirect effect of CHRP is crucial and should be given priority by reason of it can motivate individuals’ intentions, and then promote more and more behaviors.

Third, our results manifested that more PEA does not necessarily contribute to more PEB, which is contradictory to the TPB that proposes attitude can influence or determine behavior^24^. In the field of environmental protection, plentiful studies have shown that environmental attitude exerts a significant impact on PEB such as green purchases^136^, waste classification and recycling^137^, and hotel employees’ green practice^138^. At hospital, Widianto, Kautsar^19^ demonstrated that environmental attitude can explain and predict the disposal of medical wastes produced in the healthcare process. Apart from attitude related to environmental protection, there are still many factors that contribute to or impede behavior, such as environmental concern^139^, health consciousness^140^, job satisfaction^123^, and altruistic values^18^. Furthermore, hospital workers’ corporate social responsibility (CSR) is also important, which has multiple effects not only protecting human health but also reducing the carbon footprint generated by medical practices^141^. Moreover, environmental knowledge can translate into pro-environmental behavior under certain circumstances, however, this knowledge could be affected by demographic elements such as gender^25^. Additionally, the relationship between environmental attitude and PEB can be mediated or moderated by multiple elements such as organizational facilitators and barriers^17^, altruistic values^18^, and related knowledge^142^. To sum up, the influencing factor of PEB is diverse, attitude alone cannot determine actual behavior.

Fourth, we found that there is a mediating role by PEI between CHRP and PEB. Based on the TPB, intention usually is regarded as a direct determinant of behavior, when the intention is strong and beneficial, the PEB will be adopted by people^24^. Most research on PEI focuses on elucidating its direct impact on behavior, for example, Carfora, Caso^143^ used a longitudinal design approach to assess the role of intention in the prediction of actual behavior. Wu, Font^26^ demonstrated that environmental intention during holiday also makes an elusive impact on environmental behavior at home. Merely considering the direct effect is far from enough, and there is an obvious intention-behavior gap that needs further exploration, hence, the mediating role of intention becomes an emerging research topic. Consistent with past research, environmental intention is a vital mediator variable between other factors and environmental behavior, for instance, Liu, Teng^25^ proved the mediating role of environmental intention in the process of environmental knowledge translates into pro-environmental behavior. Likewise, Sabri, Razak^144^ also claimed that intention plays an important mediating role regarding to Malaysian public employees’ environmental behavior. Whether it is a direct effect or a mediating effect, we can claim that PEI is a powerful and significant predictor of PEB. However, decision-making about adopting PEB is a complicated process and is fundamentally a people process^145^. Meanwhile, this accepted fact also promulgates the reason why CHRP has an insignificant impact on PEB in our study. In conclusion, the relationship among CHRP, PEI, and PEB is strong and can be applied to predict actual behavior.

Finally, the result of multiple linear regression analysis indicated that different demographic variables have different effects on hospital workers’ CHRP, PEA, PEI, and PEB. Specifically, compared with males, females are more inclined to display more PEA and PEI, and then perform more PEB, which is consistent with the study of Swim, Gillis^146^, Ahmad, Ullah^23^, Singleton, Lau^17^, and Wei, Sial^141^. The social role theory-individuals incline to possess diverse roles and perform different behavior in the same social setting-can illustrate these differences between genders, females are more concerned about environmental issues and have a stronger willingness and belief to relieve or reverse them^23,141^. Therefore, the goal of mitigating and adapting to climate change can be a reality with the active participation of females. Moreover, we found that length of employment is meaningful, hospital workers who have worked for 6–15 years are more aware of the severity of environmental issues and adopt more PEB in their work every day. Consistent with one study to evaluate determinants of employees’ pro-environmental behavioral intentions, which takes the length of employment into consideration and demonstrate it can change intention^147^. It has become general knowledge that length of employment is positively correlated with age. According to Singleton, Lau^17^, young people tend to show more concern regarding to environmental issues and are likely to adopt more environmental protection measures. The finding resulting from the comparison of different departments is overarching, we observed higher PEA, PEI, and PEB in departments such as the department of hospital infection management, the department of prevention care, and the department of publicity. Different from clinical departments that diagnose and treat diseases, these departments protect human health through the management and prevention diseases^148^. Hence, hospital workers in these departments may possess higher PEA and PEI, and adopt more PEB. However, there are no significant differences in terms of CHRP among the three demographic groups. Up to now, although health effects from climate change are noteworthy, health risk perception is little-known, and the study related to it is also rare^149^. Therefore, it is urgent to understand the current situation of CHRP, and why it does not directly contribute to PEB at hospital. The study focusing on CHRP may help to create a new starting point for the implementation of actual PEB.

## Conclusion

The main aim of this study was to explore how climate change health risk perception (CHRP) shapes pro-environmental attitude (PEA), pro-environmental intention (PEI), and pro-environmental behavior (PEB) among hospital workers. This study presented a chain of causation from CHRP to PEB using SEM conducted by AMOS 26.0. The results indicated that CHRP has a significant positive effect on PEA and PEI, and PEI has a significant positive effect on PEB. Additionally, the indirect effect of CHRP on PEB was also significant, which was mediated by PEI. Moreover, different demographic variables comprising gender, length of employment, and employment department had different effects on PEA, PEI, and PEB.

The contributions of this study are outstanding. At the theory level, this study introduces the concept of health risk perception into the process of environmental protection to compensate for theoretical gaps, provides a new viewpoint to explore PEB, and adds new content to future research on environmental protection. Moreover, this study regarded the TPB, the ABC, and the IAB as theoretical foundation to form a comprehensive framework including CHRP, PEB, PEI, PEA, and demographic and work factors to explain PEB among hospital workers, which can better illustrate the influence of PEB and narrow the apparent attitude-intention-behavior gap at hospital. Additionally, it explained the translation from CHRP to PEB through the mediating role of PEI and PEA. In brief, current study greatly enriches the field of environmental protection.

At the practical level, the carbon footprint of hospital is obvious, this study explores the influencing factors of adopting PEB by hospital workers, transforming CHRP into PEB is important for decreasing carbon emissions. Furthermore, hospital workers’ PEB is critical to improve environmental health and public health, and meanwhile enhance workers’ satisfaction and well-being. Moreover, hospital workers are widely respected and become role models for the public, which means that a certain PEB performed by hospital workers can contribute to others to imitate and perform the same behavior. Accordingly, researchers and policy-makers should pay more attention to hospital workers and formulate appropriate interventions to promote the adoption of PEB.

This study has some limitations which require close attention in subsequent studies. First, this study only considers the effect of climate change health risk perception, other social-psychological variables, such as environmental concern, perceived effectiveness, and emotion, are not considered. In future, above mentioned critical variables should be measured together with the TPB and other related theories such as the theory of protection motivation^145^ and the theory of reasoned action^150^. Second, as cross-sectional research, this study does not reveal how to change individuals’ pro-environmental behavior. In future, we can use longitudinal methods to indicate the effectiveness of specific interventions, and then promote more pro-environmental behavior adopted by individuals. Third, the TPB is a part of the theoretical foundation of this study, however, we do not take other variables in TPB such as subjective norm and perceived behavioral control into the research model. In further research, we can integrate CHRP into complete TPB to clarify the relationship among them. Fourth, this study is conducted in Shanxi Province, China. There are wide variations of economic development situations and environmental pollution conditions among different regions, resulting in PEB may also be different from region to region. Hence, comparative studies should be conducted to explore these differences.

## Data Availability

The datasets used and/or analyzed during the current study available from the corresponding author on reasonable request.
